# Day-to-day blood pressure variability in older persons – optimizing measurement

**DOI:** 10.1097/HJH.0000000000003975

**Published:** 2025-02-20

**Authors:** Tanya Palsma, Jurgen A.H.R. Claassen, Edo Richard, Rianne A.A. De Heus

**Affiliations:** a Department of Geriatrics, Radboud University Medical Center; b Department of Geriatrics, Donders Institute for Brain, Cognition and Behaviour, Radboud University Medical Center; c Department of Neurology, Donders Institute for Brain, Cognition and Behaviour, Radboud University Medical Centre; d Department of Public and Occupational Health, Amsterdam UMC, University of Amsterdam, Amsterdam; e Department of Primary and Community Care, Radboud University Medical Center, Nijmegen, The Netherlands

**Keywords:** dementia, home blood pressure measurement, home blood pressure variability, memory clinic, memory complaints, mild cognitive impairment, optimal schedule

## Abstract

**Background:**

Higher blood pressure variability (BPV) is associated with adverse clinical outcomes but lack of standardized methodology hampers clinical translation. Day-to-day BPV seems most promising for an older population, especially those with cognitive impairment. This study aimed to determine the optimal number of measurements for obtaining day-to-day BPV in this population.

**Methods:**

We included 127 patients attending the geriatric outpatient memory clinic, who measured blood pressure for seven days, morning and evening. Blood pressure measurements of day one were discarded and the coefficient of variation was calculated to assess BPV. Concordance between 7-day BPV (CV_7days_) and a reduced number of measurement days (CV_6days_ – CV_3days_) was analysed with Bland–Altman plots, intraclass correlation coefficient (ICC), and an a priori determined threshold of a 95% confidence interval (CI) with a lower bound of 0.75.

**Results:**

The mean age was 74.6 ± 8.6 years, 49% were female, and had dementia or mild cognitive impairment in 37% and 33% respectively. Reducing the number of measurement days resulted in wider limits of agreement. Concordance decreased when reducing measurement days and reached our predefined threshold with four measurement days (ICC = 0.91, 95% CI = 0.87 – 0.93). BPV derived from five measurement days showed a similar relationship with diagnosis as our reference BPV value obtained with seven days.

**Conclusion:**

Our results suggest that systolic home blood pressure should be measured in the morning and evening for at least five consecutive days in duplicate to obtain reliable day-to-day BPV values in older adults with cognitive complaints.

## INTRODUCTION

Blood pressure variability (BPV) refers to the fluctuations in blood pressure over time. It has often been regarded as random noise affecting the estimation of mean blood pressure (BP). However, having a higher BPV has been associated with adverse clinical outcomes including, cardiovascular disease, stroke, and cognitive decline [1–3]. In older adults, BPV is proposed to hold a larger prognostic value than in younger individuals, and older age is associated with higher variability in blood pressure [4,5]. Thus, measuring and monitoring BPV in this population may be clinically relevant as it can provide prognostic information beyond mean BP.

Various types of BPV have been defined, depending on the timing and setting of the measurement (i.e., long-term: visit-to-visit, short-term: day-to-day or 24-h ambulatory). Short-term assessment has the advantage of providing a BPV value within a short time window, indicating its applicability in clinical practice. However, an at-home BP device for measuring day-to-day BPV is easier to tolerate for older adults than an ambulatory 24-h assessment [6]. Currently, there is no universal methodology to measure day-to-day BPV, which results in a high degree of heterogeneity among studies in terms of home BPV (HBPV) measuring and monitoring. This makes it difficult to compare BPV measures from different studies and complicates interpretation.

Studies investigating day-to-day BPV methodology are scarce. The European Society of Hypertension recommends using home blood pressure measurements (HBPM) standards, with at least three (preferably seven) days of morning and evening measurements [7]. No studies have explored this in older adults with cognitive complaints, leaving a knowledge gap regarding the optimal schedule in this population. For example, it is not known whether the investment of one week of measurements from patients is necessary, an argument that is particularly relevant in a population of older adults with cognitive impairment.

With the aim of reducing patient burden when assessing day-to-day BPV, we determined the optimal number of measurements to obtain home day-to-day BPV in older adults with cognitive complaints or dementia. We aimed to identify the minimum number of days of BP measurements needed to calculate day-to-day BPV without reducing its monitoring or prognostic properties. A secondary aim of this study was to investigate if day-to-day BPV can be obtained with single BP readings instead of duplicate BP readings. We validated our findings by looking into previously identified associations between BPV and dementia diagnosis [2].

## METHODS

### Study design and participants

Data for this study were derived from a convenience sample of people attending the geriatric memory clinic of the Radboud University Medical Center (Nijmegen, The Netherlands). The protocol has also been described elsewhere [8,9]. Inclusion lasted from January 2014 until July 2019. All eligible patients who visited the memory clinic were asked to perform HBPM. Eligibility was judged by the attending physician or nurse based on the capability to understand the Dutch language and to perform the BP measurements at home.

The study was submitted to the Medical Ethics Committee (CMO Arnhem-Nijmegen) and was exempt from formal approval because the study did not fall within the remit of the Dutch ‘Medical Research Involving Human Subjects Act’. The study was also exempt from the need to obtain explicit written informed consent because of the low additional burden of HBPM. Despite this, oral informed consent was asked from each participant.

### Home blood pressure measurements

Participants and their informal caregiver received instruction, both written (including visual instructions) and oral instructions by the attending physician, on how to perform HBPM. After instruction they were asked to perform a practice BP measurement to ensure they understood the device and knew how to place the cuff on the arm. Participants could be aided by their informal caregiver. Participants performed HBPM using a validated, memory-equipped, automatic oscillometric device (Microlife WatchBP Home, Microlife, Heerbrugg, Switzerland) [10]. The measurements were carried out according to an international protocol [11]. This protocol consisted of the instruction to measure BP twice a day: in the morning (4 : 00–11 : 00 a.m.) and in the evening (4 : 00–11 : 00 p.m.), for seven consecutive days. Participants received instructions to rest for five minutes before measuring BP and not to perform measurements within one hour after food or drug intake. During each measurement, two BP readings were taken automatically.

### Other variables

Additional information was extracted from the participant's medical record. Information included age, sex, smoking status, alcohol consumption, body mass index (BMI), diabetes mellitus, hypertension, office BP, and mean BP. Hypertension was defined as mean home SBP ≥135 mmHg and/or home DBP ≥85 mmHg. Office BP was measured in a supine position using a manual sphygmomanometer. All information was collected as part of standard practice during a memory clinic visit. Cognitive diagnoses were established in a multidisciplinary meeting with geriatricians and neuropsychologists based on information from the geriatric assessment using international criteria [12,13]. When deemed necessary, additional diagnostic testing (i.e., neuropsychological testing or neuroimaging) was performed. For descriptive purposes, we categorized participants into four groups: dementia (any type), mild cognitive impairment, subjective cognitive decline, and other diagnoses (i.e., psychiatric disorder).

### Statistical analysis

For all analyses, BP readings of the first day were discarded following standard HBPM protocol, resulting in 24 readings on six consecutive days. For the primary analysis, duplicate readings were averaged to reduce variability resulting from measurement error, resulting in a total of 12 readings. Day-to-day BPV was defined as the within-subject coefficient of variation (CV) for both systolic blood pressure (sBP) and diastolic blood pressure (dBP). CV was used as an indicator for BPV as it provides an accurate intra-individual estimate of BPV [14] and is relatively easy to calculate and interpret [15]. The CV is calculated by dividing the within-subject standard deviation (SD) by the mean (CV = SD/mean × 100%). We then calculated the CV with 12 averaged BP readings on six consecutive days from day two till day seven (CV_7days_), 10 BP readings from day 2 till day 6 (CV_6days_), etc., until four BP readings from day two till day three (CV_3days_). For the secondary analysis, CV was calculated in a similar manner, differing in that the first reading of each measurement was used instead of the average of both readings, resulting in CV_7daysSingle,_ CV_6daysSingle_, CV_5daysSingle_, CV_4daysSingle_, and CV_3daysSingle_. CV_7days_ was used as the reference because this is in accordance with the current practice of measuring home BP and is seen as the golden standard. Thus, CV_7days_ is seen as the ground truth of a real CV.

We analysed CV_7days_ and CV's obtained from a fewer number of measurement days with a paired t-tests and intraclass correlation coefficients. Based on the ICC guidelines from Koo and Li we deemed good to excellent reliability sufficient [16]. Therefore, a 95% confidence interval with a lower bound of at least 0.75 was sufficiently concordant with CV_7days._ Furthermore, we used Bland-Altman plots to visualize the degree of agreement between CV_7days_ and the reduced CV's based on the distribution of the 95% limits of agreement and the mean difference. Furthermore, we performed subgroup analysis, investigating if the different groups (dementia, MCI, or SCD) required a different number of measurement days.

Furthermore, to investigate the validity of the CV values obtained with fewer measurement days, we investigated the association of BPV with dementia. We used logistic regression to investigate the odds of dementia based on CV_7days_ and with fewer measurement days. In a previous study [2] with this population, we found that BPV was higher in patients with dementia compared to patients without dementia. Patients with missing cognitive diagnosis (*n* = 2) were excluded from this analysis. We did not account for specific confounders because we were specifically interested in comparing the associations and not in the association itself.

Additionally, to investigate whether measuring in duplicate is necessary, we compared the CV from our primary analysis with the CV from the same number of days with single BP readings. We analysed this with a paired *t*-test, Pearson's correlation coefficient, ICC, and Bland-Altman plots. We used the same threshold as in our primary analysis. We then also investigated if the association remained.

Finally, we investigated if antihypertensive treatment influenced our primary outcome, by analysing subgroups stratified by use of no use of antihypertensive drug classes used.

All analyses were performed using R statistical software (Version 4.1.3; R Foundation for Statistical Computing, Vienna, Austria). Statistical significance was *α* < 0.05 on two-tailed tests.

## RESULTS

### Baseline characteristics of participants

341 patients performed HBPM. Of these, 127 obtained all 28 BP measurements and were enrolled in the analyses. Table 1 shows the baseline characteristics of the study sample. Mean age of the study sample was 74.6 ± 8.6 years, and 48.8% were female. Dementia was diagnosed in 46 (36.8%), mild cognitive impairment in 41 (32.8%), 31 (24.8%) were classified as having subjective memory complaints, seven were diagnosed with another cause for their memory complaints (e.g., depression), and 2 with missing information on their cognitive status. The dementia subtypes that were diagnosed were: Alzheimer's disease (26), vascular dementia (8), mixed dementia (7), unknown etiology (4), and primary progressive aphasia (1).

**TABLE 1:** Baseline characteristics of the study sample

Characteristic	
*N*	127
Age (years)	74.6 ± 8.6
Female sex (%)	62 (48.8%)
Body mass index (kg/m^2^)	25.9 ± 3.9
Current smoker	11 (8.8%)
Alcohol use	79 (69.3%)
Diabetes mellitus	19 (15%)
Hypertension	60 (47.2%)
On antihypertensive treatment (%)	68 (53.5%)
Types of antihypertensive agents used (%)	
- ACEi	26 (20.5%)
- ARB	18 (14.2%)
- Beta-blocker	41 (32.3%)
- CCB	17 (13.4%)
- Diuretics	33 (26%)
Office systolic blood pressure (mmHg)	161.4 ± 24.5
Office diastolic blood pressure (mmHg)	84.9 ± 11.2
Mean home systolic blood pressure (mmHg)	138.4 ± 16.5
Mean home diastolic blood pressure (mmHg)	78.3 ± 9.8
Cognitive diagnosis:^a^	
- Dementia, any type	46 (36.8%)
- Mild cognitive impairment	41 (32.8%)
- Subjective cognitive impairment	31 (24.8%)
- Other^b^	7 (5.6%)

Results are presented as mean ± standard deviation or as a number (%). ACEi, ACE inhibitor; ARB, angiotensin receptor blocker; CCB, calcium channel blocker.

a *n* = 125, 2 missing due to lack of knowledge on cognitive status.

b Neurological or psychiatric diagnosis.

### CV_7days_ versus coefficient of variations for fewer measurement days

**FIGURE 1:**
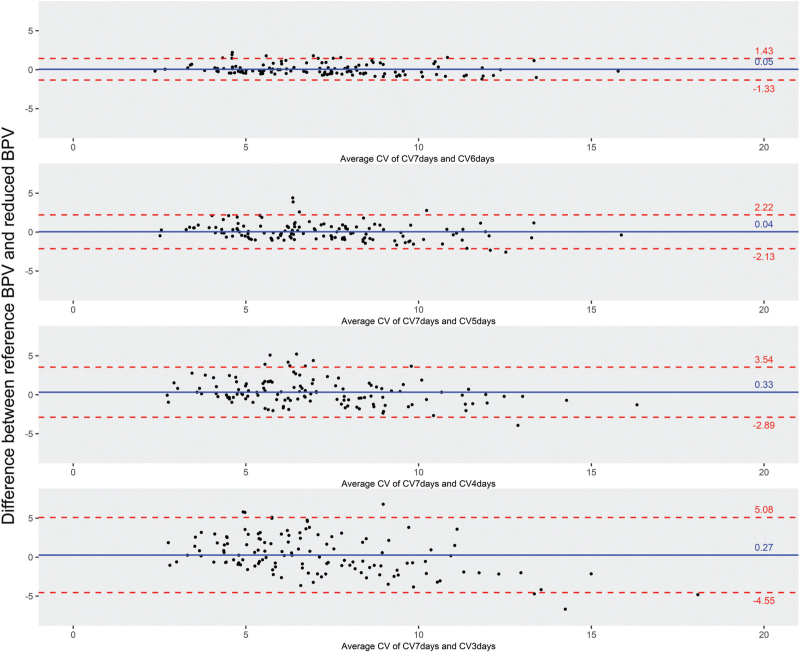
Bland-Altman plots comparing systolic CV's. The dashed lines mark the upper and lower limits of agreement; the solid line reflects the mean difference (bias) between the reference CV and a reduced CV. CV, coefficient of variation.

Measuring seven consecutive days resulted in a mean systolic CV value of 7.21 ± 2.43 and mean diastolic CV of 7.04 ± 2.58. Table 2 and Fig. 1 show the primary analysis results, illustrating a smaller CV with a decrease in the number of measurement days. CV_5days_ showed the highest concordance, while still within the threshold [for systolic: intraclass correlation coefficient (ICC) = 0.91; 95% confidence interval (CI) = 0.87–0.93]. Furthermore, CV_5days_ showed a small mean difference and narrow limits of agreement (mean difference: 0.04, *P* = 0.66, 95% CI = −0.15–0.24; limits of agreement: −2.13–2.22). Calculating the CV based on less than 5 days showed larger differences and did not fall within the threshold. Similar results were found for diastolic CV: CV_5days_ ICC = 0.81, 95% CI = 0.85–0.92; mean difference = 0.04, *P* = 0.70, 95% CI = -0.18–0.27; limits of agreement: −2.46–2.55. Visual representation of agreement for diastolic blood pressure can be found in supplementary material (Figure 1, Supplemental Digital Content). Similar results were found when investigating the subgroups (based on cognition) separately (see Table 2, Supplemental Digital Content).

**TABLE 2:** Correlation and concordance between reference BPV and reduced BPV values

	Type of CV	Mean CV	Mean difference (95% CI)	Intraclass correlation coefficient (95% CI)
Systolic	CV_7days_	7.21 ± 2.43	NA	NA
	CV_6days_	7.16 ± 2.57	0.05, (−0.07–0.18), *P* = 0.40	0.96 (0.94–0.97)
	CV_5days_	7.17 ± 2.73	0.04, (−0.15–0.24), *P* = 0.66	0.91 (0.87–0.93)
	CV_4days_	6.88 ± 2.90	0.33, (0.04–0.62), *P* = 0.03	0.81 (0.74–0.86)
	CV_3days_	6.94 ± 3.45	0.27, (−0.17–0.70), *P* = 0.22	0.66 (0.55–0.75)
Diastolic	CV_7days_	7.40 ± 2.58	NA	NA
	CV_6days_	7.44 ± 2.74	−0.04, (−0.15–0.07), *P* = 0.50	0.97 (0.96–0.98)
	CV_5days_	7.36 ± 2.94	0.04, (−0.18–0.27), *P* = 0.70	0.89 (0.85–0.92)
	CV_4days_	7.10 ± 3.04	0.31, (−0.04–0.65), *P* = 0.08	0.76 (0.68–0.83)
	CV_3days_	6.80 ± 3.78	0.60, (0.08–1.11), *P* = 0.02	0.59 (0.47–0.70)

Results are presented as mean ± standard deviation. CI, confidence interval; CV, coefficient of variation.

We found that higher systolic CV_7days_ was significantly associated with increased odds of having dementia [odds ratio (OR) 1.35, 95% CI = 1.10–1.73]. As a validation of the CV obtained with 5 days of measurement, we found a similar association between CV_5days_ and dementia with an odds ratio of 1.31 and a 95% confidence interval of 1.08–1.63. We did not find any significant association between diastolic CV and dementia.

### Duplicate or single readings: CV_5days_ vs. CV_5dayssingle_

Analysis comparing CV_5days_ from duplicate BP measurements with CV_5daysSingle_ from only the first BP reading of each measurement showed a higher CV for systolic as well as diastolic CV (systolic: mean difference = −0.60, *P* < 0.001, 95% CI = −0.85 to −0.36; diastolic: mean difference = −0.73. *P* < 0.001, 95% CI = −1.01 to −0.46) (Table 3). Furthermore, the Bland–Altman plots show relatively wide limits of agreement from −3.35 to 2.14 (see Figure 2, Supplemental Digital Content and Figure 3, Supplemental Digital Content). However, ICC values and 95% CI are within the threshold for both systolic and diastolic BPV (ICC = 0.88, 95% CI = 0.83–0.91; ICC = 0.88, 95% CI = 0.83–0.91). Analysing the association between cognition and sBPV CV_5daysSingle_ revealed that the association disappeared (OR: 1.16, 95% CI = 0.99–1.40). Additionally, comparing CV_5days_ with CV_5daysSingle_ from the second BP reading of each measurement showed that using only the second reading results in a significantly higher CV for systolic as well as diastolic CV (systolic: mean difference = −0.33, *P* = 0.004, 95% CI = −0.56 to −0.11; diastolic: mean difference = −0.52, *P* < 0.001, 95% CI = −0.79 to −0.26). However, both ICC values and 95% CI are within our threshold (ICC = 0.9, 95% CI = 0.86 – 0.93; ICC = 0.88, 95% CI = 0.84–0.92).

**TABLE 3:** Correlation and concordance between CV_5days_ vs. CV_5daysSingle_

	Type of CV	Mean CV	Mean difference (95% CI)	Intraclass correlation coefficient (95% CI)
Systolic	CV_5days_	7.17 ± 2.73	NA	NA
	CV_5daysSingle_	7.77 ± 2.97	−0.60, (−0.85 to −0.36), *P* < 0.001	0.88 (0.83–0.91)
Diastolic	CV_5days_	7.36 ± 2.94	NA	NA
	CV_5daysSingle_	8.09 ± 3.39	−0.73, (−1.01 to −0.46), *P* < 0.001	0.88 (0.83–0.91)

Results are presented as mean ± standard deviation. CI, confidence interval; CV, coefficient of variation.

### Antihypertensive medication

54% of participants used antihypertensive treatment. Use versus nonuse of antihypertensive medication did not increase the number required measurement days (Table 3, Supplemental Digital Content).

## DISCUSSION

This study investigated the minimum number of measurements and number of days of measurement needed to obtain home day-to-day BPV in older adults who attended our memory clinic for cognitive evaluation. We found strong to excellent concordance between our reference sBPV value, obtained using seven days of measurements, and the sBPV value calculated from duplicate BP measures using five consecutive days. Furthermore, the observation of a higher sBPV in patients with dementia could still be identified using sBPV value obtained using five instead of seven consecutive days. Similar results were observed for dBPV; however, no difference in dBPV in patients with dementia was evident using either seven or five days.

We further assessed the effect of reducing the number of BP readings by using single measurements, the first or the second, instead of duplicate measurements, however, doing so introduced more inaccuracy. We additionally showed that the use of AHD did not increase the number of measurement days that are minimally necessary to assess day-to-day BPV.

Studies on the optimal HBPM schedule to assess day-to-day BPV are scarce. In the Finn-Home study, a minimum of HBPM during three days was necessary to establish day-to-day BPV and accurately predict cardiovascular risk. Extending monitoring to seven days showed minimal improvement [17]. However, this study differs from ours in several aspects. First, a population of healthy adults (mean age of 57 years) was studied, whereas we investigated an older population with memory complaints, known for an increased BP and BPV. A second difference is that the Finn-Home study used a longitudinal design investigating the association between BPV and increased cardiovascular risk, whereas we used a cross-sectional design to study the association between BPV and cognition.

Our study investigated older adults with cognitive complaints because BPV has been associated with dementia and cognitive impairment [2]. This association is especially relevant in older adults since they have more fluctuations in their blood pressure [4,5,18,19]. Additionally, they have a higher risk of developing Alzheimer's Disease or cognitive impairment caused by other diseases, and a higher BPV further increases this risk. However, longitudinal data may inform us about the potential causal relationship between BPV and cognition or show reverse causality. Further research should focus on this causality and investigate if it is possible to reduce BPV. If so, measuring and monitoring BPV is important in this population to assess, monitor, and hopefully reduce cognitive risks.

Our study has several strengths and limitations. Although participants (and in most cases an informal caregiver) received instructions on how to perform HBPM correctly, they performed them unguided, making it impossible to ensure adherence to instructions. However, using a validated memory-equipped device with a preprogrammed measurement schedule limits user error and reporting bias. A strength of the current study is the method used to try and enhance adherence. In this study an informal caregiver without cognitive disorders was present during the office visit – including the HBPM demonstration and practice – and during the measurements week. We used the most effective way to ensure proper HBPM performance, of teaching the patient how to perform the HBPM themselves [20]. Despite this, only 127 patients from the total 341 patients (37%) completed all 28 BP measurements needed to be eligible. This may show that assessing day-to-day BPV by measuring HBP for seven consecutive days is too burdensome or difficult. However, 251 patients (74%) performed at least 80% of the BP measurements, showing that HBPM is feasible in this population. We did not find any differences between the patients who successfully performed all 28 measurements and those who did not (see Table 1, Supplemental Digital Content). We did not ask information about who (participant or caregiver) performed the HBPM. We expect that many patients with dementia received help from a caregiver, which may have positively affected the outcome and feasibility of HBPM as this represents the real-life situation. Another limitation is that patients with lower cognitive functioning were less likely to agree to perform HBPM, leading to selection bias, as shown in an earlier study in this population [9]. However, our study sample still contained a large diversity in cognitive functioning. Furthermore, because patients were only asked to participate if the attending physician deemed them capable to perform the HBPM, our study sample may not be fully representative of all older adults with cognitive complaints.

This study is the first to investigate the optimal schedule to assess day-to-day BPV in a population of older adults with cognitive complaints. Improved knowledge concerning the optimal schedule could have several clinical implications for the future. If further research shows that BPV can be reduced, day-to-day BPV might be used more in predicting and monitoring cognition, cardiovascular diseases or mortality. Furthermore, an optimal, universal schedule would allow us to compare BPV values. Our results are a first step in reducing patient burden and improving the clinical practicality of day-to-day BPV.

In conclusion, we found that day-to-day BPV can be assessed by measuring HBP in duplicate in the morning and evening on five consecutive days in older people attending the memory clinic. Thus, we recommend measuring BP on five consecutive days instead of seven consecutive days when assessing day-to-day BPV to minimize the burden in patients with memory complaints, MCI or dementia.

## ACKNOWLEDGEMENTS

We are grateful to all participants and their caregivers for their efforts. We would like to thank the secretary and all involved residents of the Radboudumc Alzheimer Center for their support with the recruitment and gathering of data. This study was funded by the Dutch Alzheimer Society (grant number WE.09-2015-03). Current study is a recipient of ABOARD (www.aboard-project.nl), which is a public-private partnership receiving funding from ZonMW (#73305095007) and Health∼Holland, Topsector Life Sciences & Health (PPP-allowance; #LSHM20106).

Current work has not been previously presented.

T. Palsma received funding from ABOARD, which is a public-private partnership receiving funding from ZonMW Nationaal Dementieprogramma (#73305095007) and Health∼Holland, Topsector Life Sciences & Health (PPP-allowance; #LSHM20106). More than 30 partners participate in ABOARD (www.aboard-project.nl). ABOARD also receives funding from Edwin Bouw Fonds and Gieskes-Strijbisfonds.

J.A.H.R. Claassen received funding from ZonMW/NWO, the National dementia strategy funding for the MODEM consortium, for the ‘Good Vibes’ consortium from JPND/ZonMW, from Alzheimer Nederland a cofounding for the ZonMW and JPND projects. A NWO funding for the MOCIA consortium. A funding from the ABOARD consortium from ZonMW, Gieskes-Strijbisfonds and Health-Holland.

E. Richard receives funding from The Netherlands Organisation for Health Research and Development (ZonMW) for project 1051003210004

R.A.A. de Heus receives funding from NWO for the MOCIA consortium and from ZonMW, Gieskes-Strijbisfonds and Health-Holland for the ABOARD consortium.

### Conflicts of interest

There are no conflicts of interest.

## Supplementary Material

Supplemental Digital Content

## Supplementary Material

Supplemental Digital Content

## Supplementary Material

Supplemental Digital Content

## Supplementary Material

Supplemental Digital Content

## Supplementary Material

Supplemental Digital Content

## Supplementary Material

Supplemental Digital Content
