# Supplementary material for: Day-to-day blood pressure variability in older persons – optimizing measurement
Source: J Hypertens. 2025 Feb 20;43(6):970–5. doi: 10.1097/HJH.0000000000003975 (PMC12052059; doi:10.1097/HJH.0000000000003975)
Supplement: Supplemental Digital Content [file jhype-43-0970-s003.pdf]

Supplementary Figure 3: Bland-Altman plots comparing diastolic CV5days with diastolic CV5daysSingle

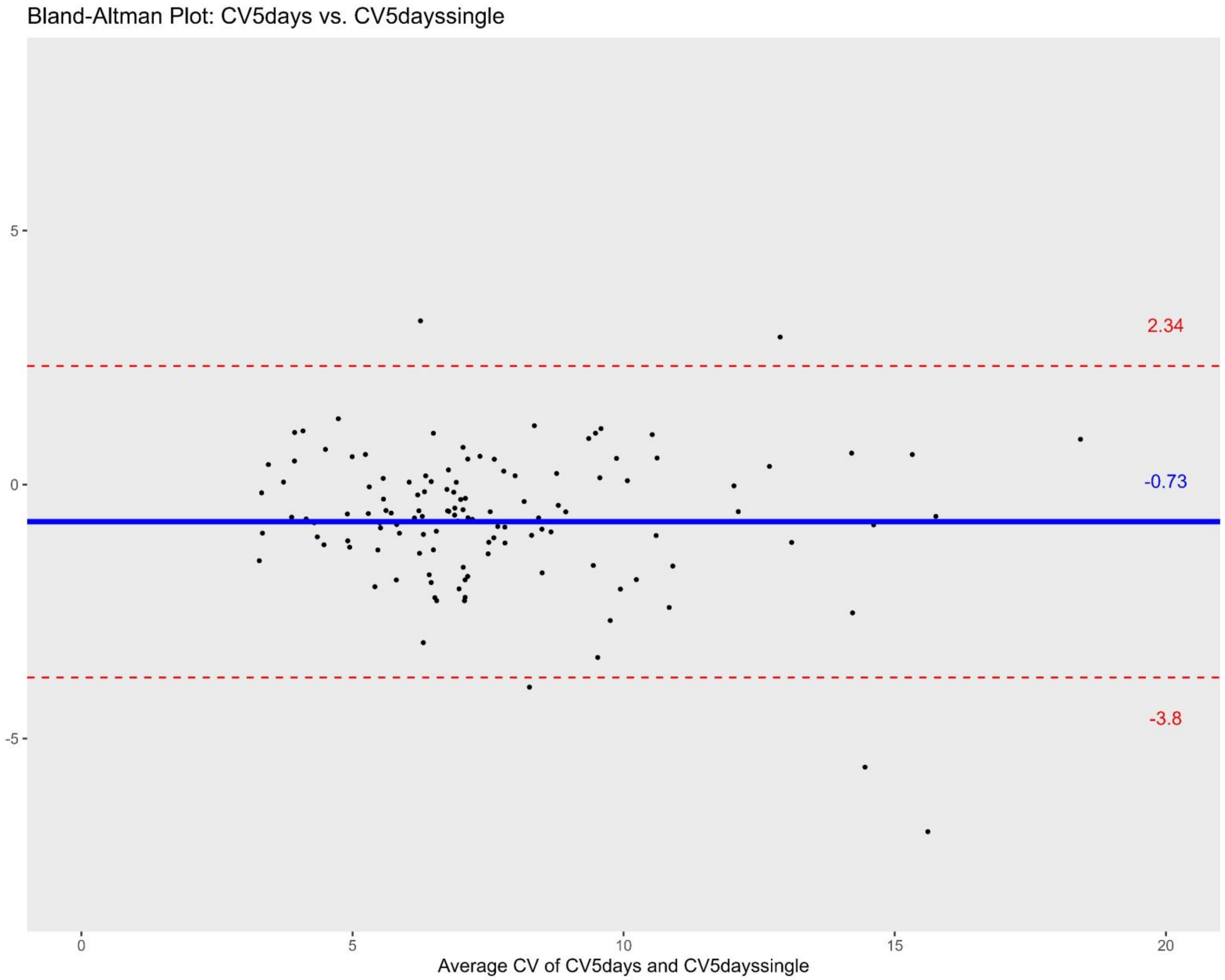

The dashed lines mark the upper and lower limits of agreement; the solid line reflects the mean difference (bias) between reference CV and a reduced CV.
