# Supplementary material for: Day-to-day blood pressure variability in older persons – optimizing measurement
Source: J Hypertens. 2025 Feb 20;43(6):970–5. doi: 10.1097/HJH.0000000000003975 (PMC12052059; doi:10.1097/HJH.0000000000003975)
Supplement: Supplemental Digital Content [file jhype-43-0970-s004.docx]

**Supplementary Table 1:** Differences in baseline characteristics between study population and rest of the sample

| Characteristic | Successful 28 BP measurements | Unsuccessful 28 BP measurements | P |
| --- | --- | --- | --- |
| N | 127 | 214 |  |
| Age in years | 74.6 ± 8.6 | 73.4 ± 9.2 | 0.218 |
| Female sex | 62 (48.8%) | 92 (43.4%) | 0.391 |
| BMI (kg/m2) | 25.9 ± 3.9 | 26.4 ± 4.4 | 0.309 |
| Current smoker | 11 (8.8%) | 31 (14.6%) | 0.125 |
| Alcohol use | 79 (69.3%) | 134 (63.2%) | 0.816 |
| Hypertension | 60 (47.2%) | 98 (46.2%) | 0.945 |
| Cognitive diagnosis:   - Dementia - MCI - SCD - Other* | 46 (36.8%)  41 (32.8%)  31 (24.8%)  7 (5.6%) | 73 (34.4%)  71 (33.5%)  47 (22.2%)  22 (10.4%) | 0.483 |

*Results are presented as mean ± standard deviation or as a number (percentage). BMI: body mass index. MCI: mild cognitive impairment. SCD: subjective cognitive impairment.
* Neurological or psychiatric diagnosis.*
