# Supplementary material for: Day-to-day blood pressure variability in older persons – optimizing measurement
Source: J Hypertens. 2025 Feb 20;43(6):970–5. doi: 10.1097/HJH.0000000000003975 (PMC12052059; doi:10.1097/HJH.0000000000003975)
Supplement: Supplemental Digital Content [file jhype-43-0970-s005.docx]

**Supplementary Table 2:** Correlation and concordance between reference BPV and reduced BPV values stratified by cognitive diagnosis

| **Type of CV** | **Mean CV** | **Mean difference**  **(95% CI)** | **Intraclass correlation coefficient (95% CI)** |
| --- | --- | --- | --- |
| **Dementia n = 46** | | | |
| CV_7days_ | 7.79 ± 2.28 | NA | NA |
| CV_6days_ | 7.71 ± 2.26 | 0.07, p = 0.51  (-0.15 – 0.29) | 0.95  (0.91 – 0.97) |
| CV_5days_ | 7.65 ± 2.53 | 0.14, p = 0.43  (-0.21 – 0.48) | 0.89  (0.80 – 0.93) |
| CV_4days_ | 7.43 ± 2.70 | 0.36, p = 0.14  (-0.21 – 0.83) | 0.79  (0.66 – 0.88) |
| CV_3days_ | 7.79 ± 3.01 | -0.003, p = 0.99  (-0.69 – 0.69) | 0.63  (0.41 – 0.77) |
| **MCI n = 41** | | | |
| CV_7days_ | 7.63 ± 2.10 | NA | NA |
| CV_6days_ | 7.64 ± 2.40 | -0.01, p = 0.93  (-0.24 – 0.22) | 0.95  (0.91 – 0.97) |
| CV_5days_ | 7.74 ± 2.45 | -0.11, p = 0.47  (-0.42 – 0.20) | 0.91  (0.84 – 0.95) |
| CV_4days_ | 7.41 ± 2.89 | 0.22, p = 0.44  (-0.35 – 0.80) | 0.74  (0.57 – 0.85) |
| CV_3days_ | 7.17 ± 3.66 | 0.46, p = 0.33  (-0.48 – 1.41) | 0.49  (0.22 – 0.69) |
| **SCD n = 31** | | | |
| CV_7days_ | 6.11 ± 2.75 | NA | NA |
| CV_6days_ | 5.64 ± 2.89 | 0.17, p = 0.18  (-0.08 – 0.43) | 0.97  (0.94 – 0.99) |
| CV_5days_ | 5.86 ± 2.98 | 0.26, p = 0.26  (-0.20 – 0.71) | 0.91  (0.81 – 0.95) |
| CV_4days_ | 5.72 ± 2.90 | 0.39, p = 0.18  (-0.19 – 0.98) | 0.84  (0.70 – 0.92) |
| CV_3days_ | 5.59 ± 3.59 | 0.53, p = 0.16  (-0.23 – 1.28) | 0.79  (0.61 – 0.89) |

Results are presented as mean ± standard deviation
