# Supplementary material for: Day-to-day blood pressure variability in older persons – optimizing measurement
Source: J Hypertens. 2025 Feb 20;43(6):970–5. doi: 10.1097/HJH.0000000000003975 (PMC12052059; doi:10.1097/HJH.0000000000003975)
Supplement: Supplemental Digital Content [file jhype-43-0970-s006.docx]

**Supplementary Table 3:** Correlation and concordance between reference BPV and reduced BPV values stratified by use of antihypertensive drugs.

| Type of CV | Mean CV | Mean difference  (95% CI) | Intraclass correlation coefficient (95% CI) |
| --- | --- | --- | --- |
| No AHD | | | |
| CV_7days_ | 6.64 ± 2.12 | NA | NA |
| CV_6days_ | 6.62 ± 2.14 | 0.02, p = 0.82  (-0.13 – 0.17) | 0.96  (0.94 – 0.98) |
| CV_5days_ | 6.55 ± 2.25 | 0.09, p = 0.51  (-0.18 – 0.36) | 0.89  (0.82 – 0.93) |
| CV_4days_ | 6.16 ± 2.45 | 0.48, p = 0.04  (0.03 – 0.94) | 0.71  (0.55 - 0.81) |
| CV_3days_ | 6.05 ± 2.70 | 0.59, p = 0.07  (-0.05 – 1.22) | 0.50  (0.28 – 0.67) |
| AHD | | | |
| CV_7days_ | 7.70 ±2.58 | NA | NA |
| CV_6days_ | 7.62 ± 2.83 | 0.08, p = 0.40  (-0.11 – 0.28) | 0.96  (0.93 – 0.97) |
| CV_5days_ | 7.70 ± 3.00 | 0.00, p = 0.98  (-0.28 – 0.29) | 0.91 (0.86 – 0.95) |
| CV_4days_ | 7.51 ± 3.13 | 0.19, p = 0.30  (-0.18 – 0.57) | 0.86  (0.78 – 0.91) |
| CV_3days_ | 7.14 ± 3.84 | -0.01, p = 0.97  (-0.61 – 0.58) | 0.72  (0.58 – 0.82) |
